# Efficacy evaluation of albumin-bound paclitaxel combined with carboplatin as neoadjuvant chemotherapy for primary epithelial ovarian cancer

**DOI:** 10.1186/s12905-022-01794-y

**Published:** 2022-06-11

**Authors:** Huan Wang, Lingyun Fan, Xia Wu, Yimin Han

**Affiliations:** Master of Medicine, Department of Gynaecology, Harbin Medical University Cancer Hospital, No. 150, Haping Road, Harbin, Heilongjiang 150081 People’s Republic of China

**Keywords:** Albumin-bound paclitaxel combined with carboplatin, Neoadjuvant chemotherapy, Epithelial ovarian cancer, Efficacy evaluation

## Abstract

**Objective:**

This study aimed to compare the efficacy of albumin-bound paclitaxel combined with carboplatin (Nab-TC) with that of traditional solvent-based paclitaxel combined with carboplatin (TC) as neoadjuvant chemotherapy (NAC) regimens for primary epithelial ovarian cancer.

**Methods:**

Eighty patients with advanced primary epithelial ovarian cancer admitted for treatment at the Harbin Medical University Cancer Hospital from January 2015 to January 2020 were retrospectively selected. All patients underwent surgery after 1–4 courses of NAC with Nab-TC or TC regimen. Among the patients included for study, 40 patients in each group.

**Results:**

The ORR in Nab-TC group was better compared to TC group (45% vs 40%), but the difference was not significant (P = 0.651). While the reduction rate of CA-125 value in the Nab-TC group was significantly better (P < 0.05). The postoperative complication rate such as postoperative blood transfusion (5% vs 35%) and postoperative infusion of human albumin (25% vs 55%) were significantly lower relative to the TC group. The median progression-free survival of the Nab-TC group was significantly longer relative to the TC group (20 months vs 13 months, *P* = 0.012), and the patient’s quality of life was also better in the Nab-TC group (*P* < 0.05). Our study demonstrated that Nab-TC regimen and R0 represented the independent prognostic factors.

**Conclusion:**

The efficacy of the Nab-TC regimen as NAC for advanced primary epithelial ovarian cancer was non-inferior to that of the TC regimen along with a lower incidence of adverse reactions, a longer PFS and a higher quality of life, supporting its therapeutic value in the clinic.

## Background

Among gynecological malignancies, the incidence of ovarian cancer is the second and the mortality rate is the first. Due to its asymptomatic presentation and lack of effective screening methods, tumors in more than 75% of patients will have spread outside the pelvis region at the time of diagnosis, which is an advanced clinical stage. The main goal for advanced ovarian cancer is satisfactory cytoreductive surgery. In cases where this is not achieved, intermittent debulking surgery (IDS) following NAC has been accepted as an alternative therapeutic strategy for advanced ovarian cancer [1]. NAC is considered for patients with stage III to IV cancer presenting large tumors for whom satisfactory cytoreductive surgery is not adequately achieved (residual lesions ≤ 1 cm) or those at greater surgical risk [2]. NAC and IDS is non-inferior to primary debulking surgery (PDS) and few adverse events as well as a high optimal debulking surgery rate were observed with NAC [3, 4]. The National Comprehensive Cancer Network (NCCN) practice guidelines recommend that all intravenous drug regimens for first-line postoperative adjuvant chemotherapy for advanced epithelial ovarian cancer can additionally be used as NAC. Paclitaxel combined with carboplatin is considered the standard first-line chemotherapy regimen for advanced ovarian cancer, but solvent-based paclitaxel, a semi-synthetic drug, is a polyoxyethylated castor oil solvent that forms micelles to wrap paclitaxel. The formulation is prone to triggering allergic reactions and glucocorticoid pretreatment is often thus required before its application [5].

Compared with traditional solvent-based paclitaxel, albumin-bound paclitaxel(ABP) combines hydrophobic paclitaxel and human serum albumin carrier, which decomposes more readily in the body and effectively transports paclitaxel to tumor tissues via endocytosis. Moreover, albumin as the carrier of paclitaxel can achieve higher drug concentration in physical strength, thereby bringing higher efficacy [6]. Allergic reactions to this formulation are rare and glucocorticoid pretreatment prior to clinical application is not required. The infusion time is short and therefore highly convenient as a clinical regimen.

Food and drug administration (FDA) approved ABP in 2005 for use in metastatic breast cancer that failed combined chemotherapy as well as breast cancer that recurred within 6 months after chemotherapy [7]. In addition, ABP combined with carboplatin is established as the first-line therapy for locally advanced or metastatic non-small cell lung cancer, and ABP combined with gemcitabine is also the first-line treatment for metastatic pancreatic cancer. ABP has significant anti-tumor activity and controllable toxicity in the treatment of recurrent platinum-resistant primary epithelial ovarian cancer or peritoneal cancer [8–10]. In recent years, numerous studies showed that ABP applied to breast cancer NAC can significantly improve the pathological complete remission rate and toxicity may be tolerated compared with solvent-based paclitaxel, which might lead to ABP became the preferred choice for the treatment of primary breast cancer instead of solvent-based paclitaxel [11, 12]. In our study, we have compared the efficacy of Nab-TC and TC regimens as NAC for epithelial ovarian cancer to ascertain the advantages of albumin binding and establish the utility of paclitaxel as therapy for ovarian cancer.

## Patients and methods

The study was approved by the Institutional Review Board of Harbin Medical University Cancer Hospital. In the end, the 376 patients with ovarian cancer who underwent NAC in the Affiliated Tumor Hospital of Harbin Medical University from January 2015 to January 2020 passed the following exclusion criteria, and finally 80 patients were included in this study. All patients signed the informed consent.

### Exclusion criteria

1. Non-epithelial ovarian cancer (such as ovarian sex cord mesenchyme or germ cell malignancies et.al) were excluded(21 patients);
2. Patients with NAC other than TC and Nab-TC regimens (237 patients);
3. Patients who received other regimens because of side effects to paclitaxel or disease progression were excluded in this study (11 patients);
4. Eastern Cooperative Oncology Group(ECOG) performance status (PS) was > 2 (5 patients);
5. Patients treated with bevacizumab combined during NAC (2 patients);
6. Patients who had undergone more than four courses of NAC without surgery (10 patients);
7. Patients who lost to follow up or lacked important data (10 patients).

### Inclusion criteria

We identified a total of 80 patients who were eligible for inclusion in the study, 40 patients in each group. Including stage IV patients with metastases to the liver, spleen or lungs, but no gastrointestinal metastases and stage III patients with metastases involving the roots of the mesentery, the omentum in a pie-like shape, the mass constricted lodged in the rectal fossa or closely related to the colorectum or retroperitoneal lymph node metastasis closely related to peripheral blood vessels, which were based on CT scanning at least one measurable lesion according to the RECIST criteria.

Satisfactory IDS was defined to include total hysterectomy, bilateral salpingo-oophorectomy, omentectomy, appendectomy, and remove the visible lesions of the abdomen, pelvis and peritoneum. For patients with organ involvement, resection of the corresponding organs should be performed, such as part of the intestine, stomach, bladder, and spleen. The lymph nodes that were positive before NAC as well as the swollen lymph nodes explored during the operation (although the preoperative imaging evaluation was negative) should be performed, but remaining patients did not need to perform conventional retroperitoneal lymphadenectomy. Recurrence after NAC with IDS treatment was defined as pelvic, intraperitoneal, retroperitoneal and distant metastasis confirmed by computed tomography (CT), magnetic resonance imaging (MRI) or positron emission tomography (PET-CT) with or without an increase in the value of CA-125. Before NAC, the pathological biopsy of the pelvic tissues under the guidance of ultrasound was used to confirm the diagnosis of cancer, and the specific pathological types were further clarified based on the postoperative pathology. The number of chemotherapy courses and the delay of chemotherapy in both groups were recorded. The incidence of postoperative complications was assessed by the following aspects: the incidence of postoperative blood transfusion, albumin infusion, postoperative venous thromboembolism and serious postoperative infection, and by calculating the postoperative hospital stay to assess the speed of postoperative recovery of patients.

### Treatment methods

The therapeutic dose of ABP for the Nab-TC group was 260 mg/m^2^ with instillation for 30 min. The patient did not undergo anti-allergy pretreatment and there was no need to monitor vital signs during the infusion process. The traditional solvent-based paclitaxel regimen required desensitization treatment prior to application including oral dexamethasone 20 mg at 12 h before treatment, oral dexamethasone 20 mg at 6 h before treatment, and intravenous cimetidine 300 mg at 60 min before treatment and iphenhydramine 50 mg was injected intramuscularly at 30 min before treatment. The treatment dose range was 135–175 mg/m^2^. Overall, 30 mg of traditional paclitaxel was dissolved into 100 mL of 0.9% sodium chloride injection and instilled slowly. The patient was observed for the first 30 min, and after no abnormal reaction was evident, the remaining drug was dissolved in 500 mL of 0.9% sodium chloride for injection. A static point was maintained for 3 h and vital signs were monitored throughout the process. Carboplatin was administered intravenously on the second day. The area under the carboplatin curve (AUC) was 5. The carboplatin dose (mg) was calculated as AUC (usually 5 mg/mL/min) × [creatinine clearance (mL/min) + 25] and female creatinine clearance rate as [(140-age) × weight (kg) × 1.23] × 0.85/serum creatinine (µmol/L). To prevent vomiting and nausea during platinum infusion, granisetron hydrochloride 30 mg was intravenously administered prior to chemotherapy. Every course of treatment, the effects were evaluated.

### Evaluation standards

The clinical tumor size and nodal status were evaluated by CT or MRI. CT scans were used to detect distant metastases. The tumor size was monitored by the same method (CT or MRI) at protocol completion. After completion of each chemotherapy regimen, the patient underwent assessment for blood tumor index CA-125 as well as gynecological MRI or CT examination. Patients with pleural effusion or lung metastasis were subjected to chest CT. Data were clinically classified into the following progression disease (PD) groups: new lesions appearing with an increased rate of ≥ 20%: stable disease (SD); tumor enlargement < 20%, tumor reduction < 30%: partial remission (PR); tumor reduction ≥ 30% with a duration of more than 4 weeks: complete remission (CR), whereby the mass disappeared completely for more than 4 weeks. The objective remission rate (ORR) was calculated as (PR + CR)/total number of cases × 100%.

PS was evaluated using the ECOG criteria. The quality of life (QOL) of the patients was followed up by telephone and scored by the European Organization for Research and Treatment of Cancer QLQ-C30 (V3.0) questionnaire. According to the criteria of WHO for evaluating subacute and acute toxicity of anti-tumor drugs, adverse reactions to chemotherapy were classified as grades I to IV. Bone marrow suppression after chemotherapy was evaluated by following up the blood indexes of patients after chemotherapy. Pain, peripheral nerve paresthesia, nausea and vomiting were assessed after chemotherapy, along with allergies and other adverse reactions.

Residual tumor size, operation time, intraoperative blood loss and the rate of performing lymphadenectomies as well as resection of important organs at IDS were extracted from surgical records. We evaluated the degree of anemia and low protein according to the postoperative check-up sheet, and obtained the incidence of postoperative blood transfusion and albumin through the doctor's order sheet. The CA-125 value before and after NAC treatment was extracted from the testing report to calculate the decrease rate. Progression-free survival (PFS) was calculated from the first start date of NAC to disease progression or the deadline for follow-up. The overall survival (OS) is calculated from the first NAC start date to death or the deadline for follow-up.

### Statistical analysis

SPSS 26.0 software was used for statistical analysis. Measurement data were expressed as mean ± standard deviation (x ± SD).Counting data is expressed as number of cases and rate (%).The independent sample *t*-test was used for comparison of continuous variables and chi-square for comparison of categorical variables between groups. The survival curve was constructed using Kaplan–Meier method (R version 4.0.5) and log-rank test to detect differences between groups. Univariate and multivariate analysis for PFS were performed using the Cox proportional hazards model. Moreover, Hazard ratios (HR) and 95% confidence intervals (95%CI) were estimated. Data were considered significant at *P* < 0.05.

## Results

Among the 376 patients with advanced primary ovarian cancer who received NAC treatment in our hospital, 80 cases were included in this study, all of whom were women. No significant difference in age at diagnosis between Nab-TC group and TC group (53.7 ± 7.532 vs 55.45 ± 6.504 *P* = 0.269), and the median age was 53 years of two groups (range 44–70 years). We classified age by 53 to evaluate its relationship with prognosis (Table 1). The BMI in Nab-TC group was 24.16 ± 5.159 and 24.16 ± 5.159 in TC group, which was no significant difference.

**Table 1:** Comparison of characteristics of the two groups

Characteristic	Nab-TC (n = 40)	TC (n = 40)	Statistics	*P* value
*Number (percent)*				
Age (years)			χ^2^ = 1.805	0.179
> 53	16 (40)	22 (55)		
≤ 53	24 (60)	18 (45)		
PS			χ^2^ = 0.000	1.000
0	16 (40)	16 (40)		
1	24 (60)	24 (60)		
FIGO stage			χ^2^ = 0.346	0.556
III	34 (85)	32 (80)		
IV	6 (15)	8 (20)		
CA-125 value (U/mL)			χ^2^ = 0.800	0.371
> 672	18 (45)	22 (55)		
≤ 672	22 (55)	18 (45)		
> 100	40 (100)	38 (95)		0.494
≤ 100	0 (0)	2 (5)		
Pathological type			–	1.000
Serous cystadenocarcinoma	40 (100)	40 (100)		
Mean number ± SD				
BMI	24.16 ± 5.159	23.04 ± 3.495	t = −1.140	0.258
NAC cycles	2.25 ± 0.899	2.05 ± 0.986	t = −0.948	0.346
Total cycles	6.35 ± 1.122	7.00 ± 0.906	t = 2.851	0.006

*PS:* Performance status, *BMI*: body mass index, SD: Standard deviation, *FIGO*: International Federation of Obstetrics and Gynecology, *CA-125:* Carbohydrate Antigen 125

The deadline for follow-up is January 1, 2021, and the median observation period was 25 months (range 13–67 months). All patients were assessed by CT scan and the pathological tissue was punctured by pathological biopsy under ultrasound guidance before chemotherapy to confirm the diagnosis of cancer. In our study, included only one pathological types according to the postoperative pathology: high-grade serous cystadenocarcinoma of the ovary (no endometrioid carcinoma, mucinous carcinoma, and clear cell carcinoma et.al). Besides, there  were no significant differences in PS (included 0 and 1 in our study) and FIGO stages of the two groups before initial treatment. The median value of CA-125 before the initial treatment of the two groups was 672 U/mL (43.7–19,451 U/mL). Based on the research of Rodriguez et al.[13], we selected 672U/mL and 100U/mL as the cut-off values for preoperative CA-125 levels to see whether it correlated with the effect of NAC as well as PFS. The evaluation showed that there was no difference in CA-125 levels before NAC between the two groups (Table 1). The difference in the number of NAC cycles (Nab-TC vs TC: 2.25 ± 0.899 vs 2.05 ± 0.986 *P* = 0.346) between two groups was not to a significant extent, but the total number of chemotherapy cycles was significantly longer in TC group (6.35 ± 1.122 vs 7.00 ± 0.906 *P* = 0.006). Two groups of patients did not change chemotherapy regimens after surgery.

The CR, PR, SD, and PD of Nab-TC group and TC group after NAC treatment were 0,18,22,0 vs 1,15,23,1, and the ORR of the two groups (Nab-TC group vs TC group was 45% vs 40% *P* = 0.651) was no significant difference (Table 2). The percentage reduction of CA-125 after NAC (including the two groups with a reduction ratio greater than 90% and 95%) and the proportion of CA-125 value < 100U/mL after NAC were significantly higher in the Nab-TC group compared to the TC group (*P* < 0.05).There was no statistical difference in the degree of satisfactory cytoreductive surgery(R0 in Nab-TC group vs TC group was 25% vs 20% *P* = 0.592)between the two groups and neither group underwent bowel resection, but one patient in the Nab-TC group underwent splenectomy. Five patients in the TC group performed lymphadenectomies and three in the Nab-TC group. The intraoperative blood loss and postoperative hospital stay in the Nab-TC group were all less than those in the TC group (*P* < 0.05), but there was no statistically significant difference in the operation time. In addition, the incidence of postoperative blood transfusion and postoperative albumin transfusion was lower in the Nab-TC group was significantly lower than that in the TC group (*P* < 0.05).

**Table 2:** Comparison of efficacy indicators (n%)

Efficacy index	Nab-TC (n = 40)	TC (n = 40)	Statistics	*P* value
*Number (percent)*				
Residual disease at IDS			χ^2^ = 0.287	0.592
R1	30(75)	32(80)		
R0	10(25)	8(20)		
Postoperative blood transfusion	2(5)	14(35)	-	0.001
Postoperative infusion of human albumin	10(25)	22(55)	χ^2^ = 7.500	0.006
Percentage drop of CA-125 value				
> 90%	26(65)	8(20)	χ^2^ = 16.573	0.000
> 95%	14(35)	6(15)	χ^2^ = 4.267	0.039
< 100U/mL	28(70)	12(30)	χ^2^ = 12.800	0.001
ORR	18(45)	16(40)	χ^2^ = 0.205	0.651
Mean number ± SD				
Operation time (min)	135.75 ± 41.333	139.65 ± 46.853	t = 0.395	0.694
Intraoperative blood loss (mL)	147.5 ± 120.336	208.0 ± 142.383	t = 2.053	0.043
Postoperative hospital stay (day)	6.9 ± 1.780	8.9 ± 4.062	t = 2.852	0.006
QOL				
Functional areas score	36.88 ± 2.766	39.63 ± 2.404	t = 4.746	0.001
Symptom areas score	32.03 ± 2.537	35.60 ± 3.209	t = 5.527	0.001
General health areas score	8.25 ± 1.373	7.25 ± 1.171	t = −3.505	0.001

R1: diameter of residual lesion < 1 cm, R0: no macroscopic residual tumor, ORR: objective remission rate, QOL: quality of life

There was no venous thromboembolism and no serious postoperative infection in the two groups. The QOL questionnaire was completed by the patients themselves or their family members.in our study. For functional areas and symptom areas, the higher the score, the worse the QOL, while the general health status is the opposite. The QOL of patients in the Nab-TC group was significantly better after the NAC treatment compared to the TC group (*P* < 0.05 Table 2), which was based on no significant differences in the PS of patients before the initial treatment as well as the number of NAC cycles between the two groups (Table 1).

The total incidence of bone marrow suppression, liver and kidney dysfunction, allergic reactions, arrhythmia and hair loss and the incidence of grade III and IV were not statistically different between the two groups. The incidence of grade III and IV of peripheral neuropathy in the Nab-TC group was higher than that in the TC group (*P* = 0.008), but the total incidence was not statistically different. The total incidence of nausea and vomiting and the incidence of grade III and IV in the TC group were higher than those in the Nab-TC group (*P* < 0.05 Table 3). Besides, when the patients whose blood glucose was higher than normal before the initial treatment were excluded, the incidence of hyperglycemia in the TC group was higher than that in the Nab-TC group (*P* = 0.043).

**Table 3:** Incidence of adverse reactions induced by the two chemotherapeutic regimens (n%)

Adverse reactions	Nab-TC (n = 40) (n)%		TC (n = 40) (n%)		*P* value	*P* value
	I–IV	III–IV	I–IV	III–IV	I–IV	III–IV
Neutropenia	26 (65.0)	12 (30.0)	29 (72.5)	16 (40.0)	0.469	0.348
Anemia	15 (37.5)	2 (5.0)	16 (40.0)	5 (12.5)	0.818	0.235
Thrombocytopenia	7 (17.5)	3 (7.5)	6 (15.0)	1 (2.5)	0.762	0.305
Peripheral neuropathy	30 (75.0)	18 (45.0)	22 (55.0)	7 (17.5)	0.061	0.008
Nausea and vomiting	16 (40.0)	4 (10.0)	35 (87.5)	15 (37.5)	0.000	0.004
Liver dysfunction	4 (10.0)	–	7 (17.5)	–	0.330	–
Kidney dysfunction	2 (5.0)	–	3 (7.5)	–	0.644	–
Allergic reaction	1 (2.5)	–	1 (2.5)	–	1.0	–
Arrhythmia	4 (10.0)	–	7 (17.5)	–	0.330	–
High blood sugar	2 (5.0)	–	8 (20.0)	–	0.043	–
Hair loss	40 (100)	–	40 (100)	–	1.0	-

Nab-TC: albumin-bound paclitaxel combined with carboplatin, TC: solvent-based paclitaxel combined with carboplatin

**Fig. 1:**
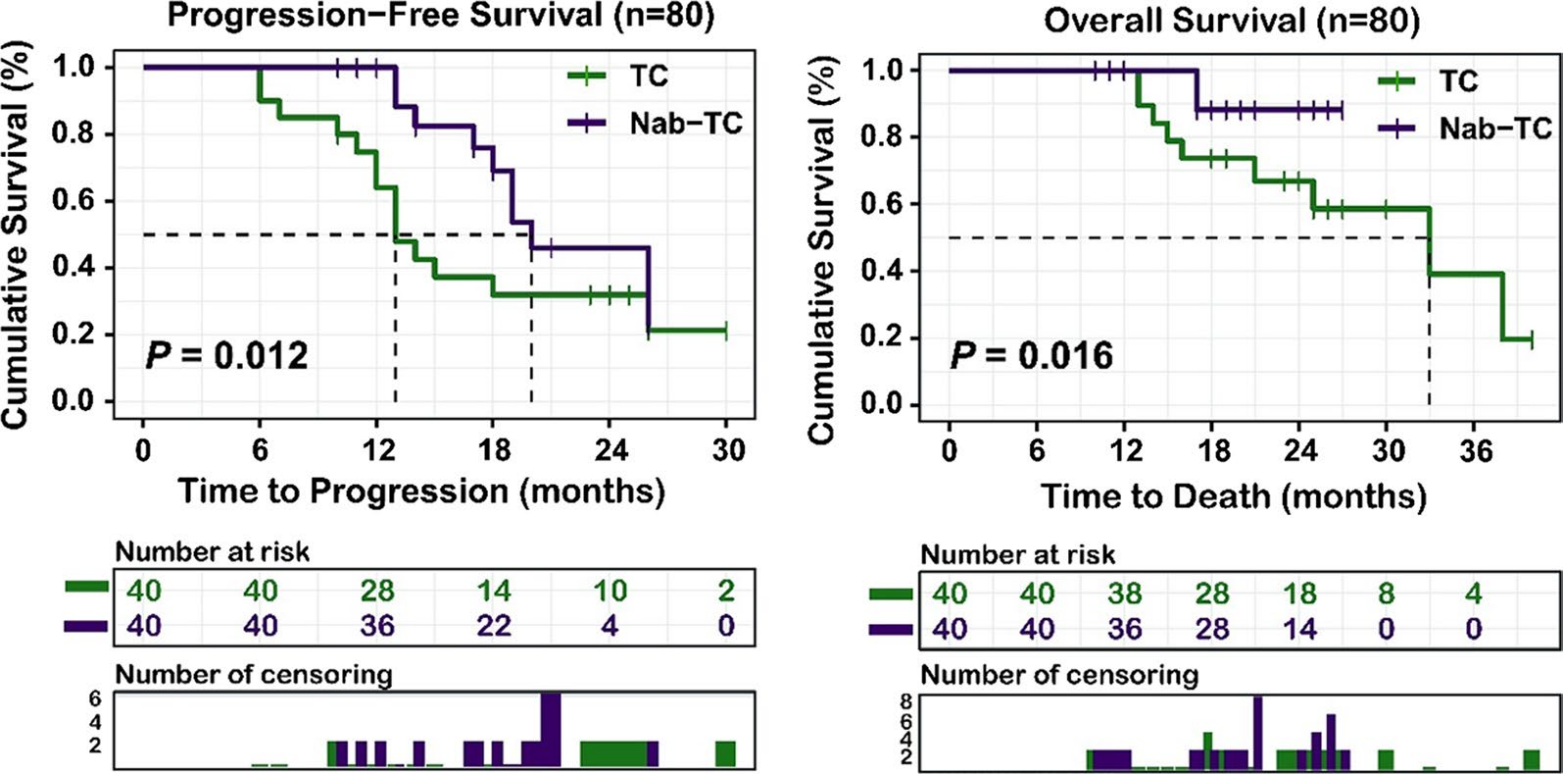
Survival outcomes between two groups

There was no significant difference in the ratio of the number of patients who actually progressed between the two groups (Nab-TC group vs TC group: 20 vs 22 *P* = 0.654), which was comparable. The average PFS of the Nab-TC group and the TC group was 21 ± 0.915 months (95%CI 19–22 months) vs 17 ± 1.383 months (95%CI 14–19 months), and the median PFS was 20 months (95%CI 16–23 months) vs 13 months (95%CI 11–14 months). The PFS of the Nab-TC group was significantly longer than that of the TC group (*P* = 0.012). Since the number of true deaths in the Nab-TC group were less than that in the TC group (4 vs 12 *P* = 0.025), the OS of the two groups was incomparable. Figure 1 showed that the survival trend of the Nab-TC group was better than that of the TC group and longer follow-up time was required to assess the difference in OS between the two groups. Our study showed that the achievement of R0 (HR = 0.180, 95%CI (0.064–0.509), *P* = 0.001) as well as the application of Nab-TC regimen (HR = 0.411, 95%CI (0.224–0.753), *P* = 0.004) were positively correlated with PFS in univariate and multivariate analysis (Table 4).

**Table 4:** Prognostic factors in the COX proportional Hazards Model

Variables	Risk ratio	Univariate 95%CI	*P* value	Risk ratio	Multivariate 95%CI	*P* value
*Age (years)*						
≤ 53/ > 53	1.768	(0.981–3.187)	0.058			
*FIGO stage*						
III/IV	1.518	(0.838–2.751)	0.168			
*PS*						
0/1	0.832	(0.450–1.537)	0.557			
*NAC cycles*						
1,2/3,4	1.522	(0.799–2.900)	0.202			
*CA-125 value (U/mL)*						
≤ 670/ > 670	0.748	(0.406–1.380)	0.354			
≤ 100/ > 100	0.770	(0.182–3.258)	0.722			
*Residual disease*						
R0/R1	0.207	(0.073–0.588)	0.003	0.180	(0.064–0.509)	0.001
*BMI*						
≤ 23/ > 23	1.518	(0.838–2.751)	0.168			
*CA-125 drop rate*						
≥ 90%/ < 90%	0.743	(0.408–1.354)	0.332			
≥ 95%/ < 95%	0.565	(0.261–1.222)	0.147			
≤ 100/ > 100U/mL	0.728	(0.405–1.309)	0.289			
*NAC regimens*						
Nab-TC/TC	0.492	(0.271–0.894)	0.02	0.411	(0.224–0.753)	0.004

FIGO: international federation of obstetrics and gynecology, PS: performance status, NAC: neoadjuvant chemotherapy, IDS: intermittent debulking surgery, CA-125: carbohydrate Antigen 125, BMI: body mass index, R1: diameter of residual lesion < 1 cm, R0: no macroscopic residual tumor

As shown in the figure, the PFS of the Nab-TC group was significantly longer compared to the TC.

group (*P* < 0.05), and the overall survival trend of the Nab-TC group was better too (*P* < 0.05).

## Discussion

Here, we evaluated the efficacy of the Nab-TC regimen as neoadjuvant chemotherapy for ovarian cancer. To this end, the age, FIGO stage, pathological type, BMI and NAC cycles compared between groups treated with Nab-TC and TC regimens were not to a significant extent, but the PFS of the Nab-TC group was significantly longer compared to the TC group. Our study demonstrated that compared with patients with R0, patients with R1 had an increased risk of progression (HR = 0.180; 95% CI 0.064–0.509; *P* = 0.001). Winter et al. [14] showed that compared with R0, patients with residual lesions of 1 cm and above had an increased risk of recurrence and death. In our operation, we performed the lymphadenectomies which were considered for metastasis before NAC as well as the swollen lymph nodes explored during the operation (although the preoperative imaging evaluation was negative) and did not perform conventional retroperitoneal lymphadenectomy. Research showed that for patients with advanced ovarian cancer with clinically negative lymph nodes, systematic pelvic and paraaortic lymphadenectomy did not have a better survival outcome compared with no lymphadenectomy and also increased the incidence of postoperative complications [15].

The optimal NAC cycle is still controversial. Colombo et al.[16] evaluated that for patients with advanced epithelial ovarian cancer, the prognosis of NAC after more than four cycles was poor. However, the study of Da Costa Miranda et al. [17] showed that for patients with stage IIIC/IV ovarian cancer, six cycles of NAC with paclitaxel combined with carboplatin were safe and effective, and the incidence of postoperative complications did not increased. In our study, to exclude the effect of NAC number on survival, we excluded patients with NAC cycles more than 4, and we found that the number of cycles of NAC (1,2 vs 3,4 *P* = 0.202) has nothing to do with PFS, and whether OS related need further follow-up data.

CT scan and CA-125 value play a recognized role in diagnosis during patient follow-up and monitoring response after treatment. Many studies tried to correlate the preoperative CA-125 levels with surgery satisfaction, PFS and OS outcomes. A Research by Mahdi et al. [18] showed that at least 90% decrease in CA-125 level after NAC is related to more complete IDS and reduced incidence of bowel resection but does not improve survival results. Rodriguez et al. [13] found that preoperative CA125 levels less than 100 U/mL are very likely to be cytoreduced to no serious residual disease. Therefore, the CA-125 value after NAC decreased by more than 90% or the preoperative CA-125 cut-off value < 100 U/mL might help to evaluate the effect of chemotherapy and determine the best timing of surgery to improve the survival. In our study, the CA-125 value decreased by > 90% and > 95% in the Nab-TC group were significantly greater than that of the TC group (65% vs 20%, *P* < 0.001; 35% vs 15%, *P* = 0.039).

ABP, a new preparation bound to human albumin, has stronger anti-tumor ability and less toxic effects compared with traditional paclitaxel preparations [19]. In addition, ABP has a relatively large volume of distribution after intravenous injection and is more widely distributed outside the blood vessels and in tissues, which achieves a high total clearance and a remarkably lowered incidence rate of treatment-related adverse reactions in comparison with conventional paclitaxel injection [20]. In this study, the incidence of hyperglycemia in the TC group was higher than that in the Nab-TC group (*P* = 0.043). In addition, the quality of life of patients in the Nab-TC group was significantly better relative to the TC group. ABP does not require glucocorticoid pretreatment and no allergic reactions before clinical administration, and reduces the incidence of hyperglycemia caused by long-term oral glucocorticoids, which is benefit to the elderly diabetic patients [21]. The clinical medication infusion time is short, which improves the patient's quality of life and chemotherapy tolerance.

Nabholtz et al. [22] reported that at a single dose of ordinary paclitaxel of 135–175 mg/m^2^, the incidence of sensory neurotoxicity was 46–70% and that of sensory neurotoxicity to degree 3 or 4 was 3–7%. This finding may be related to the demyelination and neuronal degeneration caused by organic solvents and polyoxyethylene castor oil. At an increased dose of paclitaxel to 200–250 mg/m^2^, the incidence of degree 4 sensory neurotoxicity was determined as 9–12%. In our study, the incidence of acral numbness was higher in the Nab-TC group, which could be attributable to the larger dose. Furthermore, ABP resulted in quicker recovery from peripheral neuropathy than solvent Paclitaxel [6].

## Conclusions

The efficacy of Nab-TC regimen was non-inferior to that of TC regimen, but the Nab-TC regimen had a longer PFS time, less and milder adverse reactions, better surgical tolerance and the higher quality of life. In summary, for patients with primary epithelial ovarian cancer, the clinical efficacy of the Nab-TC regimen neoadjuvant chemotherapy was better relative to the traditional TC regimen, and it was worthy of wider application and further promotion.

## Limitations and deficiencies

Our findings were limited by the small sample size and lack of long-term follow-up. Further prospective and randomized controlled trials with larger sample sizes and long-term analyses are warranted. Furthermore, relevant studies on chemotherapy resistance was lacking and there is a lack of long-term follow-up to determine the difference in overall survival between the two treatment options.

## Data Availability

The datasets generated and/or analysed during the current study are not publicly available due [medical records in the hospital could be used for scientific studies, but sharing of data was not allowed] but are available from the corresponding author on reasonable request.

## Abbreviations

ABP: Albumin-bound paclitaxel
Nab-TC: ABP combined with carboplatin
TC: Solvent-based paclitaxel combined with carboplatin
NAC: Neoadjuvant chemotherapy
IDS: Intermittent debulking surgery
PDS: Primary debulking surgery
NCCN: National Comprehensive Cancer Network
FDA: Food and drug administration
CT: Computed tomography
MRI: Magnetic resonance imaging
PET-CT: Positron emission tomography
PD: Progression disease
SD: Stable disease
PR: Partial remission
CR: Complete remission
ORR: Objective remission rate
PFS: Progression-free survival
OS: Overall survival
ECOG: Eastern cooperative oncology group
BMI: Body Mass Index
CA-125: Carbohydrate Antigen 125
PS: Performance status
SD: Standard deviation
FIGO: International Federation of Obstetrics and Gynecology
R0: No macroscopic residual tumor
R1: Diameter of residual lesion < 1 cm
QOL: Quality of Life

## References

[1] Lecointre L, Velten M, Lodi M (2020). Impact of neoadjuvant chemotherapy cycles on survival of patients with advanced ovarian cancer: a French national multicenter study (FRANCOGYN). Eur J Obstet Gynecol Reprod Biol.

[2] Wright AA, Bohlke K, Armstrong DK (2016). Neoadjuvant chemotherapy for newly diagnosed, advanced ovarian cancer: Society of Gynecologic Oncology and American Society of Clinical Oncology Clinical Practice Guideline. J Clin Oncol.

[3] Kehoe S, Hook J, Nankivell M (2015). Primary chemotherapy versus primary surgery for newly diagnosed advanced ovarian cancer (CHORUS): an open-label, randomised, controlled, non-inferiority trial. Lancet.

[4] Elies A, Rivière S, Pouget N (2018). The role of neoadjuvant chemotherapy in ovarian cancer. Expert Rev Anticancer Ther.

[5] Fader AN, Rose PG (2009). Abraxane for the treatment of gynecologic cancer patients with severe hypersensitivity reactions to paclitaxel. Int J Gynecol Cancer.

[6] Gradishar WJ, Tjulandin S, Davidson N (2005). Phase III trial of nanoparticle albumin-bound paclitaxel compared with polyethylated castor oil-based paclitaxel in women with breast cancer. J Clin Oncol.

[7] Miele E, Spinelli GP, Miele E (2009). Albumin-bound formulation of paclitaxel (Abraxane ABI-007) in the treatment of breast cancer. Int J Nanomed.

[8] Coleman RL, Brady WE, McMeekin DS (2011). A phase II evaluation of nanoparticle, albumin-bound (nab) paclitaxel in the treatment of recurrent or persistent platinum-resistant ovarian, fallopian tube, or primary peritoneal cancer: a Gynecologic Oncology Group study. Gynecol Oncol.

[9] Tillmanns TD, Lowe MP, Walker MS, Stepanski EJ, Schwartzberg LS (2013). Phase II clinical trial of bevacizumab with albumin-bound paclitaxel in patients with recurrent, platinum-resistant primary epithelial ovarian or primary peritoneal carcinoma. Gynecol Oncol.

[10] Tomao F, D'Incalci M, Biagioli E (2017). Restoring platinum sensitivity in recurrent ovarian cancer by extending the platinum-free interval: Myth or reality?. Cancer.

[11] Untch M, Jackisch C, Schneeweiss A (2016). Nab-paclitaxel versus solvent-based paclitaxel in neoadjuvant chemotherapy for early breast cancer (GeparSepto—GBG 69): a randomised, phase 3 trial. Lancet Oncol.

[12] Xie F, Chen R, Zhang L (2019). Efficacy of two-weekly nanoparticle albumin-bound paclitaxel as neoadjuvant chemotherapy for breast cancer. Nanomedicine (London).

[13] Rodriguez N, Rauh-Hain JA, Shoni M (2012). Changes in serum CA-125 can predict optimal cytoreduction to no gross residual disease in patients with advanced stage ovarian cancer treated with neoadjuvant chemotherapy. Gynecol Oncol.

[14] Winter WR, Maxwell GL, Tian C (2007). Prognostic factors for stage III epithelial ovarian cancer: a Gynecologic Oncology Group Study. J Clin Oncol.

[15] Harter P, Sehouli J, Lorusso D (2019). A Randomized Trial of lymphade ectomy in patients with advanced ovarian neoplasms. N Engl J Med.

[16] Colombo PE, Labaki M, Fabbro M (2014). Impact of neoadjuvant chemotherapy cycles prior to interval surgery in patients with advanced epithelial ovarian cancer. Gynecol Oncol.

[17] Da CMV, de Souza FA, Dos AC (2014). Neoadjuvant chemotherapy with six cycles of carboplatin and paclitaxel in advanced ovarian cancer patients unsuitable for primary surgery: Safety and effectiveness. Gynecol Oncol.

[18] Mahdi H, Maurer KA, Nutter B, Rose PG (2015). The impact of percent reduction in CA-125 levels on prediction of the extent of interval cytoreduction and outcome in patients with advanced-stage cancer of Mulleria Norigin treated with neoadjuvant chemotherapy. Int J Gynecol Cancer.

[19] Desai N, Trieu V, Yao Z (2006). Increased antitumor activity, intratumor paclitaxel concentrations, and endothelial cell transport of cremophor-free, albumin-bound paclitaxel, ABI-007, compared with cremophor-based paclitaxel. Clin Cancer Res.

[20] Liao JB, Swensen RE, Ovenell KJ (2017). Phase II trial of albumin-bound paclitaxel and granulocyte macrophage colony-stimulating factor as an immune modulator in recurrent platinum resistant ovarian cancer. Gynecol Oncol.

[21] Yamamoto Y, Kawano I, Iwase H (2011). Nab-paclitaxel for the treatment of breast cancer: efficacy, safety, and approval. Onco Targets Ther.

[22] Nabholtz JM, Gelmon K, Bontenbal M (1996). Multicenter, randomized comparative study of two doses of paclitaxel in patients with metastatic breast cancer. J Clin Oncol.

